# Scientific Authorship Through the Lens of Parenthood

**DOI:** 10.1111/1471-0528.70279

**Published:** 2026-06-10

**Authors:** Pierpaolo Nicolì, Federica Bellussi, Suneet P. Chauhan, Vincenzo Berghella

**Affiliations:** ^1^ Unit of Obstetrics and Gynecology, Department of Interdisciplinary Medicine (DIM) University of Bari “Aldo Moro”, Policlinico of Bari Bari Italy; ^2^ Division of Maternal‐Fetal Medicine, Department of Obstetrics and Gynecology Sidney Kimmel Medical College of Thomas Jefferson University Philadelphia Pennsylvania USA; ^3^ Richard D. Wood Center for Fetal Diagnosis and Treatment Children's Hospital of Philadelphia Philadelphia Pennsylvania USA; ^4^ Delaware Center of Maternal‐Fetal Medicine for ChristianaCare Newark Delaware USA

**Keywords:** authorship, parenthood, scientific writing

The idea that *creating* is an act of *giving life* is as ancient as our civilisation itself. A glance at our artistic and cultural history reveals that the theme of parenthood as a metaphor for creation has crossed the centuries, assuming ever new forms and meanings.

As early as the 4th century BCE, Plato in the *Symposium* writes that “all [humans] are pregnant both in body and in soul”. Those who do not generate physical offspring, he explains, give birth to “children of the soul”, namely thoughts and works. For the philosopher, generation is an inner and intellectual act: the soul is the womb, and thoughts and works are the children it brings to light.

With Christianity's rise, this metaphor assumed a theological connotation: in the *De Trinitate*, Saint Augustine describes the *verbum mentis* as the child of the mind, in analogy with the Divine Word generated by the Father.

During the Renaissance, this idea became more human and tangible. Michelangelo described sculpture as an act of liberating the form imprisoned within the marble, like a true birth. He believed that the artist does not create, but reveals the figure already present in the block [1], just as a parent helps a potential life to come into being.

Mary Shelley, in 1818, offered another, darker, vision of the artist's parenthood: Dr. Victor Frankenstein brings to life a being who later rejects and persecutes him, reflecting the ambivalent and sometimes conflictual bond between parent and child, and highlighting the loss of control that may follow creation.

Echoes of this metaphor persist pervasively in modern and contemporary reflections on artistic creation, often conceived as a form of inner gestation requiring time before being brought into the world, as reflected in Rainer Maria Rilke's meditations on artistic maturation, Virginia Woolf's claim to Woman's intellectual generation, and Björk's vision of artworks as independent living entities [2].

Over more than two millennia, philosophers, writers and artists of every age have recognised in *their* creative process a parallel with the birth and growth of a child. Both experiences involve generation, care, risk, loss of control, and finally, detachment.

Those of us writing this essay cannot carve stone, compose poetry or paint evocative images. Yet, we are obstetricians and gynaecologists, and every day we accompany those who carry within them the heartbeat of a new life, helping it to be born and to live [3]. Alongside this, we are engaged in scientific work, where ideas are gradually shaped, tested, and refined [4]. In this sense, science, too, has a creative dimension: it gives form to observation, uncertainty and discovery.

It may not be a coincidence that we, too, reflecting on our work, have recognised in *our* creative process, in the making of research and in scientific writing, a metaphorical path not so distant from that of parenthood. In truth, it was this parallel that found us first. In our everyday academic life, we often speak of our scientific articles as “firstborns”, “latest arrivals”, or “newborns”. What we have long said half‐jokingly one day elicited a deeper reflection: in naming our work this way, we recognise a sense of attachment to what we conceive, develop, and eventually release into the world.

We acknowledge that this is our personal perspective, and that some may smile, or even flinch, at the comparison between the birth of a scientific paper and the birth of a child. Yet, it is now clear that we are not the first to associate creation, in any of its forms, with parenthood. This analogy, whether consciously or subconsciously, has inspired countless artists, poets, writers, and musicians, each in their own language, long before us.

Ultimately, there is nothing trivial in recognising that every creative act, whether a sculpture, a poem or a scientific article, is a generative act that requires time, dedication, care, and, in the end, the ability to let go of what one has helped bring into the world.

In this spirit, we have summarised in the accompanying table (Table 1) the similarities between the process that leads to the writing of a scientific article and the journey that leads to the birth of a new life. Two processes animated by the same original force: the desire to give shape and life to something that did not exist before.

**TABLE 1:** Similarities between scientific authorship and parenthood.

	Scientific authorship	Parenthood
	Conception	
1	We consider carefully whether to begin a study	We consider carefully whether to have a child
	Gestation	
2	It takes a team to complete a research project: researchers, writers, content experts, statisticians, participants	It takes a team to support a pregnancy: family, friends, and the wider community
3	The process is long, often more than 9 months	The gestation period is long, typically around 9 months
4	There are standards to follow, such as CONSORT, PRISMA, and journal guidelines	There are standards to follow, including prenatal care, evidence‐based practice, and safety measures
5	We monitor their progress carefully through analyses, draft revisions, and peer feedback	We monitor their development and growth carefully through prenatal visits and ultrasounds
6	The research process can take unexpected turns, such as changes in resources or personnel, unforeseen results, or equipment failures	Pregnancy can follow an unpredictable course, with variations in how it progresses
	Birth	
7	We celebrate milestones in research, especially presentation or publication	We celebrate milestones in pregnancy, especially the birth of the baby
	Growth	
8	Authors may have differing views on how to interpret findings	Parents may have differing views on how to raise the child
9	We have high expectations for our articles; we want them to be solid and make a difference in the world	We have high expectations for our children; we want them to be strong and make a difference in the world
10	After publication, the article requires continued care, through dissemination, presentations, and ongoing visibility	After birth, the child requires continued care, through education, activities, and ongoing support
11	We mentor younger researchers, sharing what we have learned to help the next generation grow	We pass on what we have learned, helping our children find their own path
12	We feel proud of our work and tend to speak about it whenever we can	We feel proud of our children and tend to speak about them whenever we can
	Independence	
13	Once a paper is published or a study presented, many express differing opinions about their value and execution; this can bring pride, introspection, worry, defensiveness, and many other emotions	Many express differing opinions about our children and our parenting; this can bring pride, introspection, worry, the familiar “mama or papa bear” defensiveness, and many other emotions
	Legacy	
14	Our publications may wander far from us, taking on lives of their own	Our children may wander far from us, taking on lives of their own

## Author Contributions

Conceptualisation, project administration: Vincenzo Berghella. Methodology, writing – original draft, visualization: Pierpaolo Nicolì, Vincenzo Berghella. Writing – review and editing: Pierpaolo Nicolì, Federica Bellussi, Suneet P. Chauhan, Vincenzo Berghella. Validation: All authors approved the final version of this manuscript.

## Funding

The authors have nothing to report.

## Ethics Statement

The authors have nothing to report.

## Consent

The authors have nothing to report.

## Conflicts of Interest

The authors declare no conflicts of interest.

## Data Availability

Data sharing not applicable to this article as no datasets were generated or analysed during the current study.
